# HDAC4 induces the development of asthma by increasing Slug-upregulated CXCL12 expression through KLF5 deacetylation

**DOI:** 10.1186/s12967-021-02812-7

**Published:** 2021-06-12

**Authors:** Wendi Wei, Weida Chen, Naifeng He

**Affiliations:** 1 Department of Hepatology, Taian Hospital of Traditional Chinese Medicine, Taian, 271000 People’s Republic of China; 2 Department of Geriatric Medicine, Affiliated Hospital of Shandong University of Traditional Chinese Medicine, Jinan, 250355 Shandong People’s Republic of China; 3 School of Health, Shandong University of Traditional Chinese Medicine, Jinan, 250355 Shandong People’s Republic of China

**Keywords:** HDAC4, KLF5, Slug, CXCL12, Deacetylation, Transcriptional activity, Asthma, Airway remodeling, Inflammation

## Abstract

**Background:**

Asthma is a frequently occurring respiratory disease with an increasing incidence around the world. Airway inflammation and remodeling are important contributors to the occurrence of asthma. We conducted this study aiming at exploring the effect of Histone deacetylase 4 (HDAC4)-mediated Kruppel-like factor 5 (KLF5)/Slug/CXC chemokine ligand-12 (CXCL12) axis on the development of asthma in regulation of airway inflammation and remodeling.

**Methods:**

An asthmatic rat model was induced by ovalbumin (OVA) irrigation, and determined HDAC4, KLF5, Slug, and CXCL12 expression in the lung tissues by RT-qPCR and Western blot assay. OVA was also used to induce a cell model of asthma in human BEAS-2B and HBE135-E6E7bronchial epithelial cells. The airway hyperresponsiveness (AHR), and expression of inflammatory cytokines in model mice were examined using methacholine challenge test and ELISA. The biological behaviors were measured in asthma model bronchial smooth muscle cells (BSMCs) following loss- and gain- function approaches. The interactions between HDAC4, KLF5, Slug, and CXCL12 were also detected by IP assay, dual luciferase gene reporter assay, and ChIP.

**Results:**

HDAC4 was upregulated in lung tissues of OVA-induced asthmatic mice, and inhibition of HDAC4 alleviated the airway inflammation and remodeling. HDAC4 increased KLF5 transcriptional activity through deacetylation; deacetylated KLF5 bound to the promoter of Slug and transcriptionally upregulated Slug expression, which in turn increased the expression of CXCL12 to promote the inflammation in bronchial epithelial cells and thus induce the proliferation and migration of BSMCs.

**Conclusion:**

Collectively, HDAC4 deacetylates KLF5 to upregulate Slug and CXCL12, thereby causing airway remodeling and facilitating progression of asthma.

**Supplementary Information:**

The online version contains supplementary material available at 10.1186/s12967-021-02812-7.

## Background

Asthma is regarded as the most common chronic respiratory disease, an affects people of all ages [1]. About 300 million people are estimated to suffer from asthma around the globe, and this number is still on the rise [2]. Asthma is understood to be a complicated genetic disease, which is also strongly affected by environmental factors; its increasing incidence brings a great social burden due to morbidity, loss of quality of life, and medical costs [3]. Asthma is featured by airway inflammation, hyperresponsiveness to antigens, as well as remodeling [4]. Bronchial smooth muscle cells (BSMCs) can proliferate and release cytokines, thereby resulting in the occurrence of asthma [5]. The treatment of asthma mainly relies on management with the use of corticosteroids and bronchodilators [6]. Therefore, obtaining a better understanding of the mechanisms involved in airway inflammation and BSMC proliferation is critical for the better treatment of asthma.

Histone deacetylase 4 (HDAC4), a member of class II HDACs, is capable of inducing deacetylation, and thereby exerts important effects on the regulation of gene transcription as mediated by histone acetyltransferases [7]. As previously reported, decreased HDAC4 expression inhibits airway smooth muscle cell (ASMC) proliferation [8]. Moreover, HDAC4 was revealed to be responsible for allergic inflammation in HMC-1 cells during asthma [9]. Of note, HDAC contributed to deacetylation of Kruppel-like factor 5 (KLF5) in prostate cancer cell line DU 145 [10]. Additionally, HDAC4 inhibition could acetylate KLF5 in glioma [11]. KLF5 is a transcription factor with multiple functions, including cell proliferation, differentiation, and also carcinogenesis [12]. KLF5 could facilitate the migration and proliferation of BSMCs caused by sANE-BEAS-2B-CM and -HBE-CM in areca nut-modulated airway remodeling [13]. It has been reported that KLF5 directly promotes the transcription of Slug during mammary gland development [14] and upregulates Slug protein in Hep-2 cells [15]. Furthermore, Slug can favor airway remodeling and inflammation during asthma [16]. The induction of Slug by human rhinovirus infection could in part aid in the formation of matrix protein deposition as well as lamina reticularis thickening in the airway of asthma patients [17]. Intriguingly, Slug was revealed to upregulate CXC chemokine ligand-12 (CXCL12) expression in human osteoblasts [18]. CXCL12, which belongs to the CXC chemokine subfamily, participates in inflammatory reactions [19] and is found to accelerate fibrocyte transmigration in patients with chronic obstructive asthma and in asthma patients during an acute exacerbation [20]. Furthermore, previous studies demonstrated that CXCL12-neutralizing antibodies as well as CXCL12 neutralig can prevent the development of asthma [21, 22]. Taking the aforementioned reports into consideration, we propose a hypothesis in this study that HDAC4-mediated KLF5/Slug/CXCL12 axis should affect the development of asthma. To test this hypothesis, we constructed an asthmatic mouse model and an asthma cell model to investigate the interaction among HDAC4, KLF5, Slug and CXCL12 and their effects on airway inflammation and remodeling in vivo and BSMC proliferation and migration in vitro, thereby revealing a pathway does selective vulnerability of asthma.

## Materials and methods

### Ethical approval

The present study has been approved by the ethics committee of Affiliated Hospital of Shandong University of Traditional Chinese Medicine. The animal experiments involved in this study were conducted in accordance with the Guide for the Care and Use of Laboratory Animals of the *National Institutes of Health*.

### Construction of asthmatic mouse model

A total of 36 female BALB/c mice (7-week-old) were randomly assigned to groups for sham-treatment, asthma induction with ovalbumin (OVA), or induction with OVA and further treated with short hairpin RNA-negative control (sh-NC), sh-HDAC4, sh-HDAC4 + vector, or sh-HDAC4 + CXCL12 overexpression plasmid (CXCL12) (n = 6/group). Three days before the model establishment, the mice were injected with 200 μL (2 × 10^7^ TU/mL) of sh-NC, sh-HDAC4, sh-HDAC4 + vector or sh-HDAC4 + CXCL12 via tail vein.

OVA-induced asthmatic rats were intraperitoneally injected with 0.2 mL of OVA antigen suspension [containing 25 μg of OVA; grade V; Sigma-Aldrich, St Louis, MO, USA) and 1 mg of aluminum hydroxide (Aldrich, Milwaukee, WI, USA)] in normal saline on days 0, 7, 14, and 21, respectively. On days 27, 29, and 31, the mice were placed in sealed containers and then inhaled 20 μg OVA/50 μL normal saline mist for 30 min on each day. The stimulation test was repeated twice a week within three months,. Within 24 h after the final inhalation of the mist, the mice were euthanized, intubated, and then connected with a ventilator (SCIREQ Inc., Montreal, Quebec, Canada) The airway resistance was measured via pulmonary function method, and was used as an index of airway hyperresponsiveness (AHR) [16]. Similar procedures were performed in the sham-operated mice, but the OVA antigen suspension was replaced by normal saline.

### Methacholine challenge test

A methacholine (PHR1943; Sigma-Aldrich) challenge test was used to evaluate the AHR, and inspiratory and expiratory resistances. The mice inhaled 6.25, 12.5, 25 and 50 mg/mL of methacholine aerosol dissolved in phosphate buffer saline (PBS) for 5 s at each concentration. When the previous AHR curve was restored to the baseline level before the first inhalation of methacholine, the next inhalation of methacholine was carried out, and the peak value of airway response after each inhalation of methacholine was recorded.

### Measurement of bronchoalveolar lavage fluid (BALF)

After the measurement of AHR, 1 mL sterile PBS was infused into mouse lungs through the trachea to collect BALF, and the total number of cells in the BALF sample was counted using a blood cell counter. BALF was centrifuged (7 min, 2000 rpm) to separate the supernatant and cell pellet, which was stored at – 70 ℃ for subsequent measurements. Cell precipitates were smeared on a slide for staining, and the percentage of megaphagocytes, eosinophils, lymphocytes, and neutrophils in the BALF was determined by counting 400 leukocytes in randomly selected fields of view under light microscopy.

### Enzyme-linked immunosorbent assay (ELISA)

The supernatant from the cell culture medium was centrifuged (2000 rpm, 5 min) and the obtained supernatant was collected for further analysis, and the supernatant of BALF was likewise collected from animal experiments. The levels of interleukin 4 (IL-4; M4000B), IL-5 (M5000), IL-13 (M1300CB), and transforming growth factor-β (TGF-βe; MB100B) in BALF supernatant and cell supernatant were determined using ELISA kits (R&D systems, Minneapolis, MN, USA). The optical density (OD) values were measured directly at 562 nm using an enzyme-linked immunometric meter (Thermo Fisher Scientific Inc., Waltham, Massachusetts, USA). The standard curves were delineated with the standard protein concentration as the X-axis and the OD value as the Y-axis. The concentrations of IL-4, IL-5, IL-13, and TGF-β were obtained from the standard curve based on the OD values of the sample wells.

### Hematoxylin–eosin (HE) staining

Mouse lungs were paraffin-embedded, sectioned, cleared e in xylene I and II for ten minutes each and rehydrated in a series proceeding from anhydrous ethanol I and II followed by e 95, 90, 80, and 70% alcohol (for five minutes each), followed by rinsing with distilled water. The sections were then soaked in Harris hematoxylin for 3–8 min, differentiated with 1% hydrochloric acid alcohol for several seconds, and treated with ammonia to return the blue color. The sections were counterstained with eosin for 1–3 min and then successively immersed in 95% alcohol I and II, anhydrous alcohol I and II and cleared with xylene I and II (for five minutes each). The sections were removed from the xylene, dried in air, and sealed with neutral gum. Finally, five high-power visual fields were randomly selected from each section under a microscope to detect the infiltration of inflammatory cells around the lung and bronchus.

### Immunohistochemistry

The paraffin sections of mouse lungs were rehydrated as above and then rinsed with distilled water. Next, antigen repair was conducted in microwave with sodium citrate buffer at 92—96 ℃, for 10–15 min. The sections were naturally cooled down to room temperature, treated with 0.03% Triton-X for 10 min, and sealed with normal goat serum blocking solution (C-0005, Haoran Biotechnology, Shanghai, China) at room temperature for 60 min. The sections were then incubated with primary antibody alpha smooth muscle (α-SMA; ab32575, 1:500) overnight at 4 ℃, incubated with secondary antibody (ab205718, 1:1000, Abcam, Cambridge, UK), in the dark at room temperature for 60 min, dried, and then sealed with neutral gum. Finally, sections were observed and photographed under microscope, and five high-power visual fields were randomly selected from each section to determine the enlargement and distribution of α-SMA protein, and analyze the area of smooth muscle cells around the lung tissues bronchus.

### Measurement of hydroxyproline

A total of 60 mg of mouse lung tissue from each mouse was ground into a homogenate, and 0.25 mL of 12 NHCl was added to the samples, mixed, and incubated at 110 ℃ for 16 h. Following centrifugation, 25 μL of supernatant was added to 25 μL of citrate/acetate buffer for 20 min of incubation, followed by further incubation along with 500 μL of chloramine T solution and 500 μL of Ehrlich's solution at 65 ℃ for 15 min. After the solution had cooled down, the OD values at the wavelength of 550 nm were measured, and the hydroxyproline concentration of the samples was determined according to the curve of the standard samples.

### Cell culture and transfection

Two human bronchial epithelial cells, BEAS-2B (CRL-9609) and HBE135-E6E7 (HBE, CRL-2741), were purchased from American Type Culture Collection (Manassas, VA, USA). BEAS-2B cells were cultured in the bronchial epithelial growth medium (CC-3171, Lonza, Walkersville, MD, USA). HBE cells were cultured in the serum-free keratinocyte medium (10,744,019, Invitrogen, Carlsbad, CA, USA) containing 5 ng/mL human recombinant epidermal growth factor (PHG0313, Invitrogen), 0.05 mg/mL bovine pituitary extract (02–104, Sigma-Aldrich), 0.005 mg/mL insulin (41,400,045, Invitrogen) and 500 ng/mL hydrocortisone (803,146, Sigma-Aldrich) in a 5% CO_2_ incubator (Thermo Fisher Scientific Inc.) at 37℃. Cells in the logarithmic phase were trypsinized and seeded on a 6-well plate. After 24 h of conventional culture, OVA (5 mg/mL) was used to induce the cell model of asthma at 37 ℃ for 24 h for subsequent experiments.

Overexpressed negative lentivirus vector, overexpressed lentiviruses KLF5 and CXCL12, silencing NC lentivirus sh-NC and silencing lentiviruses sh-HDAC4, sh-Slug and sh-CXCL12 were all purchased from GenePharma (Shanghai, China). After 14–16 h of culture, BEAS-2B and HBE cells were infected with sh-NC, sh-HDAC4, sh-Slug, sh-CXCL12, sh-HDAC4 + vector, sh-HDAC4 + oe-KLF5, and sh-HDAC4 + CXCL12 according to the instructions for virus infection. After 24 h of infection, OVA (5 mg/mL) was incubated with the cells with different infection at 37 ℃ for 24 h to induce inflammation. After the induction, the culture medium was renewed with normal culture medium, followed by further culture for 24 h. The supernatant of cells with different infection was collected for ELISA detection and BSMC proliferation and induction. The expression of related genes was determined in the infected cells by RT-qPCR.

### Isolation and purification of BSMCs

After the mice were euthanized, the trachea was removed and put into PBS. Afterwards, the epithelium and fibrous tissues were removed under an operating microscope. The remaining tissues were cut into small pieces and detached with collagenase I and elastase IV for 1 h at 37℃. After centrifugation at 2000 rpm for 5 min, the precipitates were washed twice with Roswell Park Memorial Institute medium (RPMI-1640) containing 10% fetal bovine serum (FBS, 10100147, Thermo Fisher Scientific Inc.). The cells were then seeded and cultured in a moist incubator. Fresh medium was renewed every three days until the cells reached confluency, and then the cells were subcultured with 0.25% trypsin-ethylenediaminetetracetic acid solution. In all experiments, 5–8th generations of BSMCs were used and cultured with Dulbecco's modified Eagle’s medium (DMEM) containing 10% FBS, 100 μg/mL streptomycin, and 100 μg/mL penicillin (Thermo Fisher Scientific Inc.) in an incubator at 37 ℃ with 5% CO_2_.

### Reverse transcription quantitative polymerase chain reaction (RT-qPCR)

Trizol reagent (15596026, Invitrogen) was applied for extracting the total RNA. Based on the instructions of the PrimeScript RT regent kit (RR047a, Takara, Kyoto, Japan), the RNA was reversely-transcribed into complementary DNA, which was then determined by RT-qPCR with fast SYBR Green PCR kit (Applied Biosystems) and ABI prism 7300 RT-PCR system (Applied Biosystems). Each well was set with three replicates. Glyceraldehyde-3-phosphate dehydrogenase (GADPH) was used as an internal reference to analyze the relative expression of the target gene by 2^−ΔΔCt^ method. The primer design is shown in Additional file 1: Table S1.

### Western blot assay

The cells were washed with PBS and then lysed with lysis buffer (P0013, Beyotime, Beijing, China). The tissues were ground in liquid nitrogen and then lysed with lysis buffer (P0013, Beyotime). The cells were incubated at 4 ℃ for 30 min, and the lysate was collected into a 1.5 mL Eppendorf tube, followed by centrifugation at 12,000 g at 4 ℃ for 15 min, with the supernatant subsequently collected. The protein concentration was determined with the use of a bicinchoninic acid (BCA) protein concentration assay kit (Beyotime). The protein loading buffer was added into the supernatant and boiled for 5 min. After that, 20 μg protein sample was transferred to a polyvinylidene fluoride (PVDF) membrane by sodium dodecyl sulfate–polyacrylamide gel electrophoresis (Millipore, Billerica, MA, USA) and blocked with 5% skimmed milk powder for 1 h. The protein was then incubated with TBST-diluted primary rabbit antibodies (purchased from Abcam) against HDAC4 (ab12172, 1: 1000), KLF5 (ab137676, 1: 1000), Ac-Lysine (ab21623, 2 μg/mL), Slug (ab27568, 1: 1000), and CXCL12 (ab9797, 1: 1000) overnight at 4℃, with GADPH antibody (ab181602, 1: 10,000) as the internal reference. The following day, the protein was further incubated with horseradish peroxidase (HRP)-labeled secondary antibody (ab205718, goat anti-rabbit, 1: 10,000) at room temperature for 1 h, with enhanced chemiluminescence development (Baoman Biotechnology Co., Ltd., Shanghai, China) subsequently conducted. The Image J software (National Institutes of Health) was used to analyze the gray value of each band.

### Immunoprecipitation (IP) assay

The interaction between HDAC4 and KLF5 was determined. Briefly, Flag-KLF5 and Myc-HDAC4 were co-transfected into HEK-293 T cells. After 72 h of transfection, the total protein was collected and quantified by BCA method. Flag antibody and immunoglobulin G (IgG) with the same property were used in IP. Flag and Myc primary antibodies were used in IB. To examine the interaction between the proteins, 1 mg of the total protein was added to 30 μL Dynabeads and 1 μg normal rabbit IgG or to 30 μL Dynabeads and anti-Flag antibody, followed by overnight incubation with at 4 ℃. After incubation, centrifugation was conducted and the precipitates were denaturated, followed by agarose gel electrophoresis to transfer the protein to a polyvinylidene fluoride membrane. In Western blot assay, primary antibodies against Flag (#14793, 1: 50, rabbit, Cell Signaling Technologies, Beverly, MA, USA), and Myc (ab32072, 5 µg/mL, rabbit antibody, Abcam Inc.) were incubated overnight at 4℃. Next, HRP-labeled secondary antibody (ab205718, goat anti rabbit, 1: 10,000, Abcam) was incubated at room temperature for 1 h, followed by enhanced chemiluminescence development (Baoman). Finally, the gray value of each band was analyzed by Image J.

### Dual luciferase reporter gene assay

The transcriptional activity of KLF5 was determined. The reporter gene plasmids with MKK7 or Slug promoter sequence were co-transfected with KLF5 overexpression or empty plasmid into HEK-293 T cells. After 72 h, the cells were collected and the protein was extracted. A luciferase detection kit (K801-200, BioVision, Mountain View, CA, USA), and a lomax20/20 luminometer fluorescence detector (Promega, Madison, WI, USA) were used to detect luciferase activity. The primer sequences of the MKK7 promoter were as follows: F: 5′-TCGAGCTCTAGGTGGCGTCATCCTT-3; R: 5′-GGGCTGATATCCAGGTTGAGGTCGA-3′; the sequences of the Slug promoter were: F: 5′-TGCGTTCCCAAACCTCACGGA-3′; R: 5′-GCCTTCCCCACAGGCTCCCT-3'.

### Chromatin immunoprecipitation (ChIP)

To detect the binding between KLF5 and Slug promoter, An EZ-Magna ChIP TMA kit (Millipore, Billerica, MA, USA) was used for ChIP assay. BSMCs in logarithmic growth were cultured with 1% formaldehyde for 10 min, and the cross-linking was terminated by treatment with 125 mm glycine at room temperature for 5 min. The cells were washed twice with precooled PBS, centrifuged at 2000 rpm for 5 min and resuspended in cell lysate to reach a final cell concentration of 2 × 10^6^ cells/200 mL. The cells were added with the mixture of protease inhibitors, followed by centrifugation at 5000 rpm for 5 min, and resuspension with nuclear separation buffer solution. After lysis in ice water bath for 10 min, the chromatin fragments at 200—1000 bp were obtained through ultrasound treatment. The supernatant (100 μL, DNA fragment) extracted from centrifugation (at 14,000 *g* and 4 ℃, for 10 min) was added to 900 μL of ChIP dilution buffer and 20 μL of 50 × pseudoisocyanine, which were respectively added with 60 μL proteinA agarose/Salmon Sperm DNA, respectively. Following another centrifugation at 4℃ and at 700 rpm for 1 min, a 20 μL portion of the supernatant was taken as an input. In the experimental group, the supernatant was added with 2 μg KLF5 (ab137676), while the NC was added with 2 μg rabbit normal IgG (ab172730, Abcam). Moreover, 60 μL proteinA agarose/Salmon Sperm DNA was added into each tube, followed by 2 h of mixing. Subsequently, the precipitates were rinsed with 1 mL of low salt buffer, high salt buffer, LiCl solution and trace element (twice). Each tube was eluted twice with 250 mL ChIP wash buffer. The promoter of Slug (F: 5′-TGCGTTCCCAAACCTCACGGA-3′; R: 5′-GCCTTCCCCACAGGCTCCCT-3′) in the complex was quantified by fluorescence qPCR.

### Cell-counting kit-8 (CCK-8) assay

BSMCs were seeded into a 96-well plate (4 × 10^3^ cells/well, 100 μL in each well). After 24 h of culture, BSMCs were cultured in conditioned medium (the supernatant of medium used to culture BEAS-2B and HBE cells) for 72 h. After the end of culture, 10 μL CCK-8 solution (5 mg/mL; prepared with PBS) was added to each well. After 4 h of incubation, the cells were shaken for 10 min, after which the OD values were measured at the wavelength of 450 nm on an enzyme-linked immunosensor.

### Transwell assay

Transwell chambers (diameter of aperture: 8 mm; Corning, NY, USA) were used to examine cell migration in vitro in a 24-well plate. The basolateral chamber was added with DMEM containing 600 mL of 20% FBS, and maintained balanced at 37 ℃ for 1 h. BSMCs after the intervention were resuspended in DMEM without FBS. Next, 3 × 10^5^ cells/mL cells were seeded into the apical chamber, and cultured at 37 ℃ with 5% CO_2_ for 24 h. Transwell chambers were taken out and washed twice with PBS, 5 min each time, and fixed with 4% paraformaldehyde for 20 min. Subsequently, staining was performed with 0.1% crystal violet or 10 min. Cotton balls were used to wipe off the cells on the surface. Five visual fields were randomly chosen under an inverted fluorescence microscope (TE2000, Nikon, Tokyo, Japan), and the number of cells passing through the chambers was counted and the average value was recorded.

### Statistical analysis

All data were analyzed with the use of the SPSS 21.0 software (IBM Corp, Armonk, NY, USA). Measurement data were presented as mean ± standard deviation from independent experiments in triplicate. Data that obeyed normal distribution and homogeneity of variance between two groups were compared using unpaired *t*-test. Data among multiple groups were compared using one-way analysis of variance (ANOVA), combined with Tukey’s post-hoc tests. Repeated measures ANOVA was performed for comparison on data at different time points among each group, combined with Bonferroni *post-hoc* tests. Classification of data was analyzed using chi-square test. A *p* value < 0.05 demonstrated statistical significance.

## Results

### Inhibition of HDAC4 attenuates airway inflammation and remodeling in asthmatic mice

**Fig. 1:**
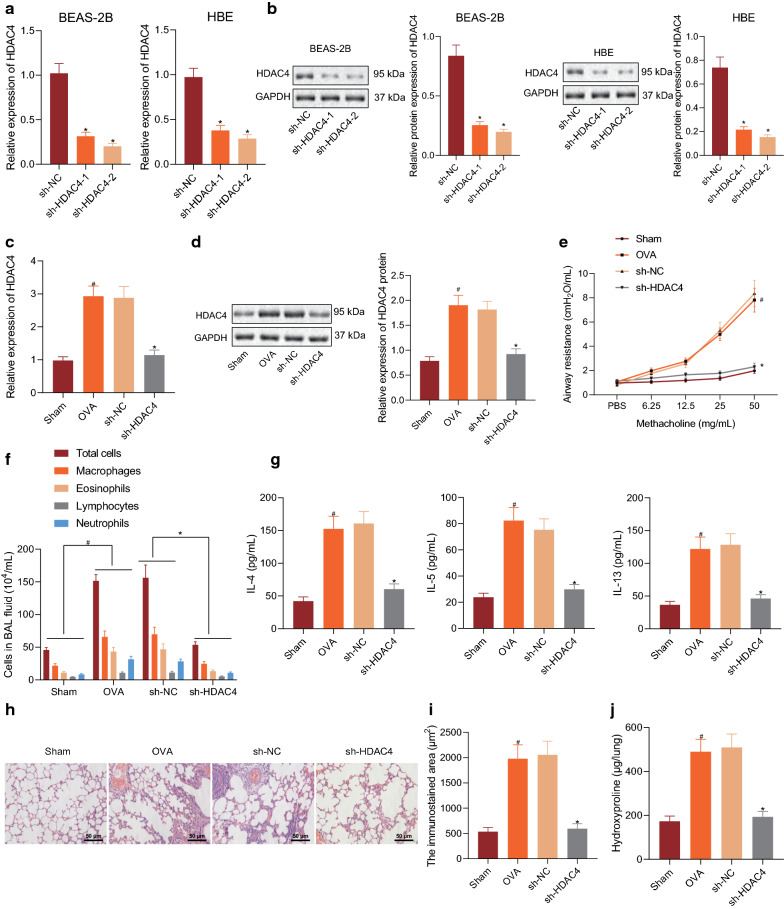
Inhibition of HDAC4 attenuates airway inflammation and remodeling in asthmatic mice. **a** The silencing efficiency of HDAC4 as determined by RT-qPCR. **b** The silencing efficiency of HDAC4 as determined by Western blot assay. **c** The expression of HDAC4 in lung tissues of mice of mice with different treatment as determined by RT-qPCR. **d** The expression of HDAC4 in lung tissues of mice with different treatment as determined by Western blot assay. **e** The AHR in mice with different treatment as examined by methacholine challenge test. **f** The number of inflammatory cells in BALF of mice with different treatment. **g** The expression of IL-4, IL-5, and IL-13 in BALF of mice with different treatment as determined by ELISA. **h** The infiltration of inflammatory cells around the pulmonary bronchus of mice with different treatment as examined by HE staining (200 × ; 50 μm). **i** The expression of α-SMA determined to measure the area of BSMCs in the lung tissues as examined by immunohistochemistry. **j** The expression of hydroxyproline determined by Western blot assay in the lung tissues of mice with different treatment determined to examine the content of collagen in the lung tissues. *p < 0.05 vs. sh-NC. # *p* < 0.05 vs. sham-operated mice. These data were measurement data, expressed as mean ± standard deviation. Data between two groups were compared using unpaired *t*-test. Data among multiple groups were compared using one-way ANOVA, combined with Tukey’s post-hoc tests. Repeated measures ANOVA was performed for comparison on data at different time points among each group, combined with Bonferroni *post-hoc* tests. n = 6

To study the role of HDAC4 in asthma, the si-HDAC4-1 and si-HDAC4-2 was transduced in human bronchial epithelial cells BEAS-2B and HBE135-E6E7 (HBE) and the silencing efficiency of HDAC4 was examined by RT-qPCR and Western blot assay; since sh-HDAC4-2 showed the best silencing efficiency (Fig. 1a, b), it was packaged into lentivirus for subsequent experimentation. The mice were sham-operated, asthma induced with OVA, or induced with OVA and further treated with sh-NC or sh-HDAC4 for subsequent animal experiments. RT-qPCR and Western blot assay showed that, compared with sham-operated mice, OVA-induced asthmatic mice exhibited an increased expression of HDAC4 in their lung tissues, while silencing of HDAC4 decreased expression of HDAC4 in lung tissues of OVA-induced mice relative to sh-NC treatment (Fig. 1c, d). Moreover, methacholine challenge test results displayed that, relative to sham-operated mice, OVA-induced asthmatic mice had increased AHR, which was inhibited by HDAC4 silencing (Fig. 1e). In addition, the number of inflammatory cells and the expression of inflammatory factors (IL-4, IL-5, and IL-13) were measured in BALF of mice with different treatments, which revealed that the number of inflammatory cells as well as the expression of IL-4, IL-5, and IL-13 in BALF of OVA-induced asthmatic mice were higher than in sham-operated mice, whereas all these indicators were normalized by sh-HDAC4 treatment (Fig. 1f, g).

As illustrated in HE staining, the infiltration of inflammatory cells around the lung and bronchus was conspicuous in OVA-induced asthmatic mice compared to sham-operated mice, which was alleviated by the sh-HDAC4 treatment (Fig. 1h). Additionally, the area of BSMCs in lung tissues was examined by determining the protein expression of α-SMA using immunohistochemistry. The results showed that, compared with sham-operated mice, the expression of α-SMA in OVA-induced asthmatic mice was increased, but was reduced in the presence of sh-HDAC4 (Fig. 1i). Hydroxyproline level in lung tissues was higher in OVA-induced asthmatic mice than in sham-operated mice. After treatment with sh-HDAC4, the expression of hydroxyproline was diminished (Fig. 1j). These results suggest that the inhibition of HDAC4 reduces airway inflammation and remodeling in asthmatic mice.

### HDAC4 promotes airway inflammation and remodeling by increasing KLF5 transcriptional activity through deacetylation

**Fig. 2:**
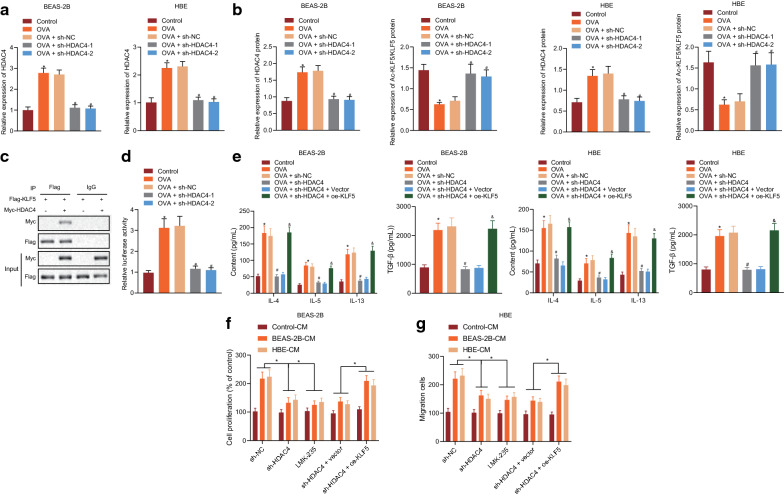
HDAC4 promotes airway inflammation and remodeling by increasing KLF5 transcriptional activity through deacetylation. **a** The expression of HDAC4 in BEAS-2B and HBE cells with different treatment as determined by RT-qPCR. **b** The acetylation level of HDAC4 and KLF5 as determined by Western blot assay. **c** The binding between HDAC4 and KLF5 as examined by IP assay. **d** The transcription activity of KLF5 as examined by dual luciferase reporter gene assay. **e** The expression of TGF-β and inflammatory factors IL-4, IL-5, and IL-13 in the supernatant of BEAS-2B and HBE cells with different treatment as examined by ELISA. **f** The proliferation of BSMCs with different treatment as examined by CCK-8 assay. **g** The proliferation of BSMCs with different treatments as examined by Transwell. *p < 0.05 *vs.* control or control-CM. #*p* < 0.05 vs. OVA + sh-NC. &*p* < 0.05 vs. OVA + sh-HDAC4 + vector. These data are measurement data, expressed as mean ± standard deviation. Data among multiple groups were compared using one-way ANOVA, combined with Tukey’s post-hoc tests

With an attempt to test the underlying mechanism of HDAC4 in the process of asthma, we infected human bronchial epithelial BEAS-2B and HBE cells with lentivirus sh-HDAC4 to inhibit the expression of HDAC4. Subsequently, BEAS-2B and HBE cells were induced with OVA to produce inflammation, and the cells were used as controls, only induced with OVA, or induced with OVA in the presence or absence of sh-NC, sh-HDAC4-1, or sh-HDAC4-2. RT-qPCR demonstrated that the expression of HDAC4 was upregulated after OVA induction but was decreased in response to sh-HDAC4 (Fig. 2a). Western blot showed that the expression of HDAC4 increased and the acetylation level of KLF5 decreased after OVA induction, while sh-HDAC4 reversed these changes (Fig. 2b).

Next, the plasmids of Flag-KLF5 and Myc-HDAC4 were transfected into HEK-293 T cells, and the binding between HDAC4 and KLF5 was examined by IP assay. The results showed that the expression of HDAC4 was present with Flag as IP, suggesting that HDAC4 could bind to KLF5 (Fig. 2c). Dual luciferase reporter gene assay was used to determine the transcriptional activity of KLF5 in cells with different treatments, results of which demonstrated that the transcriptional activity of KLF5 was elevated after OVA induction, but was decreased after treatment with sh-HDAC4 (Fig. 2d).

The results from ELISA showed that the expression of TGF-β, IL-4, IL-5, and IL-13 in the cell supernatant increased after OVA induction, while sh-HDAC reversed this change. However, further KLF5 overexpression induced an increase in TGF-β, IL-4, IL-5, and IL-13 levels in the cell supernatant (Fig. 2e). Furthermore, BSMCs were cultured with conditioned media (supernatant of the media used to culture BEAS-2B and HBE cells) control-CM, BEAS-2B-CM, and HBE-CM.. Next, CCK-8 and Transwell assays were performed to examine the proliferation and migration of BSMCs. The results demonstrated that BEAS-2B-CM and HBE-CM promoted the proliferation and migration of BSMCs. After treatment with sh-HDAC4, the proliferation and migration of BSMCs were inhibited following induction of BEAS-2B-CM and HBE-CM. LMK-235, an HDAC4 deacetylase inhibitor (2 nM, HY-18998, MCE), further suppressed the proliferation and migration of BSMCs. However, combination of HDAC4 silencing and KLF5 overexpression promoted the proliferation and migration of BSMCs (Fig. 2f, g). Overall, HDAC4 is capable of deacetylating KLF5 to increase its transcription activity, thereby promoting inflammation of bronchial epithelial cells and thus inducing proliferation and migration of BSMCs.

### KLF5 transcriptionally regulated Slug expression to promote airway inflammation and remodeling

**Fig. 3:**
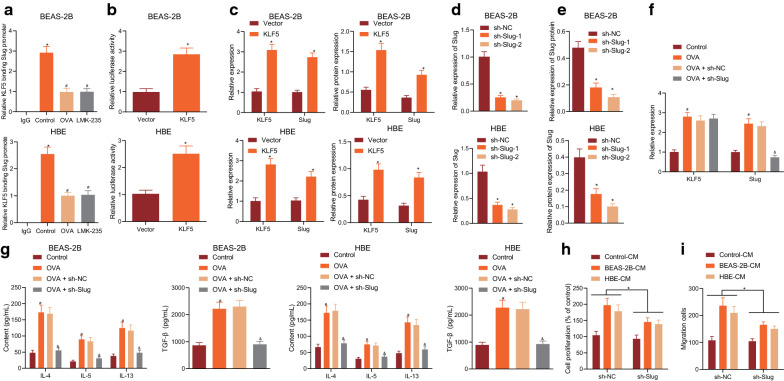
KLF5 transcriptionally regulates Slug expression to promote airway inflammation and remodeling. **a** The binding of KLF5 in the Slug promoter in BEAS-2B and HBE cells as examined by ChIP assay. **b** The KLF5 transcriptional regulation on Slug as examined by dual luciferase reporter gene assay. **c** KLF5 and Slug expression as determined by RT-qPCR and Western blot assay. **d** The silencing effect of Slug as determined by RT-qPCR. E The Slug silencing effect of Slug expression as determined by Western blot assay. **F** The expression of KLF5 and Slug in BEAS-2B and HBE cells with different treatment as determined by RT-qPCR. **g** The expression of TGF-β and inflammatory factors IL-4, IL-5, and IL-13 in supernatant of BEAS-2B and HBE cells with different treatment as examined by ELISA. **h** The proliferation of BSMCs with different treatment as examined by CCK-8 assay. **i** The migration of BSMCs with different treatment as examined by Transwell assay. *p < 0.05 vs. IgG, vector, or sh-NC. #*p* < 0.05 vs. control. These data are measurement data, expressed as mean ± standard deviation. Data between two groups were compared using unpaired *t*-test. Data among multiple groups were compared using one-way ANOVA, combined with Tukey’s post-hoc tests

Next, we explored whether KLF5 bound to the promoter of Slug to affect its transcription during asthma development. ChIP results displayed that the binding of KLF5 to the promoter of Slug was diminished in the BEAS-2B and HBE cells after the treatments with OVA or deacetylase inhibitor LMK-235 (Fig. 3a). Next, the reporter gene plasmid was co-transfected with the Slug promoter region sequence and overexpression KLF5 or empty plasmid into BEAS-2B and HBE cells, and the transcriptional regulation activity of KLF5 on Slug was examined by dual luciferase reporter gene experiment. Results showed that KLF5 promoted the promoter activity of Slug (Fig. 3b). The results of RT-qPCR and Western blot assay demonstrated that overexpression of KLF5 elevated the mRNA and protein expression of Slug (Fig. 3c). These results suggested that KLF5 could bind to the promoter of Slug to promote its expression. Moreover, sh-Slug-2 displayed the best silencing effect in HBE cells (Fig. 3d, e).

Subsequently, human bronchial epithelial cells BEAS-2B and HBE were infected with sh-Slug lentivirus. After inhibiting the expression of Slug, BEAS-2B and HBE cells were treated with OVA in the presence or absence of sh-NC or sh-Slug. The expression of KLF5 and Slug as determined by RT-qPCR were upregulated after OVA induction. After treatment with sh-Slug, the expression of KLF5 remained unchanged, while the expression of Slug was decreased (Fig. 3f). ELISA showed that the expression of TGF-β, IL-4, IL-5, and IL-13 in the cell supernatant displayed an increase after OVA induction, which was normalized in the presence of sh-Slug (Fig. 3g). Furthermore, CCK-8 assay and Transwell assay revealed that BEAS-2B-CM and HBE-CM could promote the proliferation and migration of BSMCs, while sh-Slug reversed these changes (Fig. 3h, i). These results demonstrate that KLF5 promotes the expression of Slug by binding to the promoter of Slug, and then promotes the inflammation in bronchial epithelial cells, resulting in enhanced proliferation and migration of BSMCs.

### Slug promoted the expression of CXCL12 to promote the inflammation in bronchial epithelial cells and thus induce the proliferation and migration of BSMCs

**Fig. 4:**
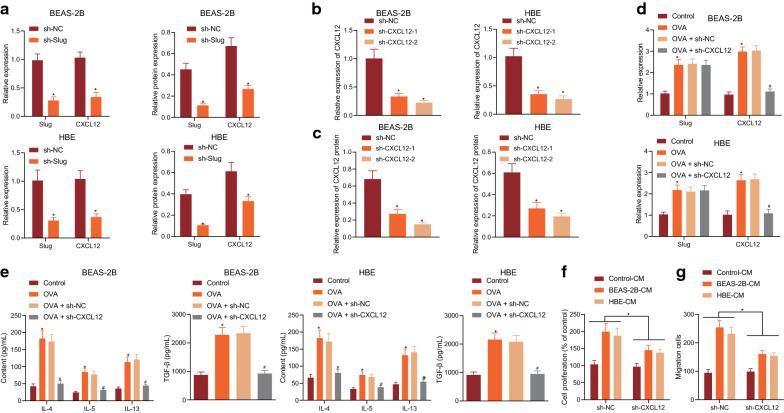
Slug promotes the expression of CXCL12 to promote tinflammation in bronchial epithelial cells and thus induce the proliferation and migration of BSMCs. **a** The expression of Slug and CXCL12 as determined by RT-qPCR. **b** The silencing effect of CXCL12 as determined by RT-qPCR. **c** The silencing effect of CXCL12 as determined by RT-qPCR. **d** The expression of Slug and CXCL12 in BEAS-2B and HBE cells with different treatments as determined by RT-qPCR. **e** The expression of TGF-β and inflammatory factors IL-4, IL-5, and IL-13 in supernatant of BEAS-2B and HBE cells with different treatments as examined by ELISA. **f** The proliferation of BSMCs with different treatments as examined by CCK-8 assay. **g** The migration of BSMCs with different treatments as examined by Transwell assay. *p < 0.05 vs. sh-NC or control. #*p* < 0.05 vs. OVA + sh-NC. These data were measurement data, expressed as mean ± standard deviation. Data between two groups were compared using unpaired *t*-test. Data among multiple groups were compared using one-way ANOVA, combined with Tukey’s post-hoc tests

Subsequently, we aimed to validate our speculation that Slug promotes the inflammation in bronchial epithelial cells and induces the proliferation and migration of BSMCs by promoting the expression of CXCL12. RT-qPCR and Western blot assay revealed that the mRNA and protein expression of CXCL12 was reduced in response to sh-Slug (Fig. 4a). Moreover, sh-CXCL12-2 had the best silencing effect in BEAS-2B and HBE cells (Fig. 4b, c). After infection of human bronchial epithelial cells with sh-CXCL12 lentivirus, BEAS-2B and HBE cells were induced with OVA to produce inflammation. Results from RT-qPCR showed that the expression of Slug and CXCL12 was upregulated after OVA induction, while the expression of Slug remained unchanged, but that of CXCL12 was reduced in the presence of sh-CXCL12 (Fig. 4d). The results from ELISA showed that OVA elevated the expression of TGF-β, IL-4, IL-5, and IL-13 in cell supernatant, which was decreased by sh-CXCL12 (Fig. 4e). As shown by CCK-8 assay and Transwell assay, BEAS-2B-CM and HBE-CM promoted the proliferation and migration of BSMCs, which were inhibited upon sh-CXCL12 treatment (Fig. 4f, g). These results demonstrate that Slug promotes the inflammation in bronchial epithelial cells and thus induces the proliferation and migration of BSMCs by promoting the expression of CXCL12.

### HDAC4 promoted the proliferation and migration of BSMCs via regulation of the KLF5/Slug/CXCL12 axis

**Fig. 5:**
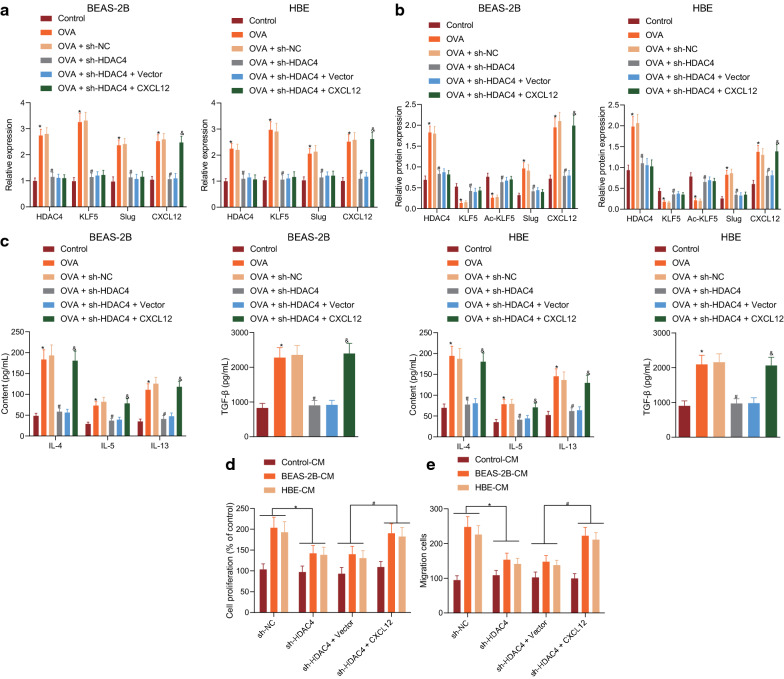
HDAC4 promotes the proliferation and migration of BSMCs via regulation of the KLF5/Slug/CXCL12 axis. **a** The expression of HDAC4, KLF5, Slug and CXCL12 as determined by RT-qPCR. **b** The expression of HDAC4, KLF5, Ac-KLF5, Slug, and CXCL12 as determined by Western blot assay. **c** The expression of TGF-β and inflammatory factors IL-4, IL-5, and IL-13 in supernatant of BEAS-2B and HBE cells with different treatment as examined by ELISA. **d** The proliferation of BSMCs with different treatments as examined by CCK-8 assay. **e** The migration of BSMCs with different treatments as examined by Transwell assay. *p < 0.05 *vs.* sh-NC or control. #*p* < 0.05 vs. OVA + sh-NC. & vs. OVA + sh-HDAC4 + vector. These data are measurement data, expressed as mean ± standard deviation. Data among multiple groups were compared using one-way ANOVA, combined with Tukey’s *post-hoc* tests

In order to further study the regulatory mechanism of HDAC4 on the KLF5/Slug/CXCL12 axis, human bronchial epithelial cells BEAS-2B and HBE were infected with sh-HDAC4 and CXCL12 lentiviruses. RT-qPCR showed that the expressions of HDAC4, KLF5, Slug, and CXCL12 were upregulated after OVA induction, but were reduced by sh-HDAC4. Compared with the sh-HDAC4, the sh-HDAC4 and oe-CXCL12 treatment contributed to increased CXCL12 expression, but failed to change the expression of HDAC4, KLF5, and Slug (Fig. 5a). Western blot assay demonstrated that the expressions of HDAC4, Slug, and CXCL12 were upregulated, and the expressions of KLF5 and Ac-KLF5 were downregulated by OVA induction. However, after treatment with sh-HDAC4, the expressions of HDAC4, Slug, and CXCL12 showed declines, while those of KLF5 and Ac-KLF5 were up-regulated. Compared with sh-HDAC4 treatment, the expression of HDAC4, KLF5, Ac-KLF5, and Slug in CXCL12 cells remained unchanged in response to sh-HDAC4 and oe-CXCL12 treatment, while the expression of CXCL12 had a notable increase (Fig. 5b).

As shown by ELISA, the expression of TGF-β, IL-4, IL-5, and IL-13 in the cell supernatant increased after OVA induction, but decreased after treatment with sh-HDAC4. sh-HDAC4 and oe-CXCL12 treatment contributed to increased expression of TGF-β, IL-4, IL-5, and IL-13 (Fig. 5c). CCK-8 and Transwell assays revealed that the proliferation and migration of BSMCs were promoted after culture of BEAS-2B-CM and HBE-CM. sh-HDAC4 treatment resulted in decreased proliferation and migration of BSMCs, which could be reversed by sh-HDAC4 and oe-CXCL12 treatment (Fig. 5d, e). Overall, HDAC4 promotes the proliferation and migration of BSMCs by regulating the KLF5/Slug/CXCL12 axis.

### HDAC4 promoted airway inflammation and remodeling in asthmatic mice via regulation of KLF5/Slug/CXCL12 axis

**Fig. 6:**
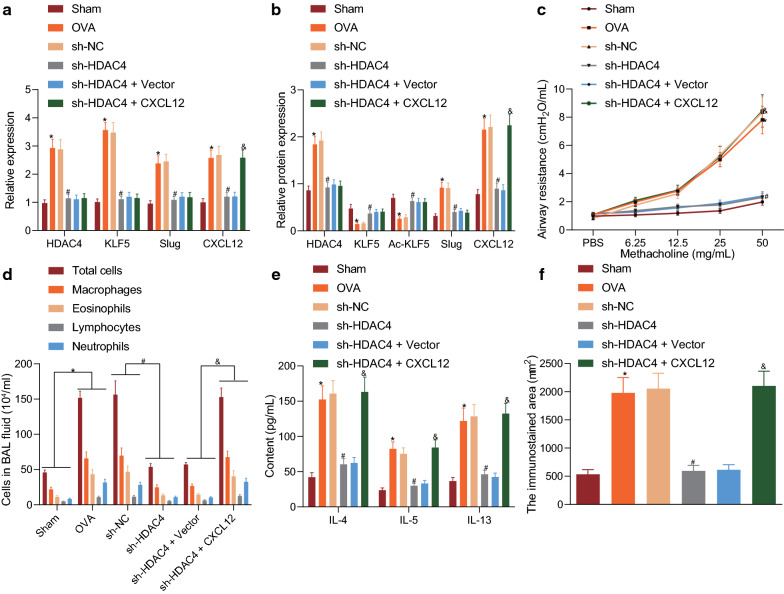
HDAC4 promotes airway inflammation and remodeling in asthmatic mice via regulation of the KLF5/Slug/CXCL12 axis. **a** The expression of HDAC4, KLF5, Slug, and CXCL12 in the lung tissues of mice with different treatments as determined by RT-qPCR. **b** The expression of HDAC4, KLF5, Ac-KLF5, Slug, and CXCL12 in the lung tissues of mice with different treatments. **c** The AHR in mice with different treatments as examined by methacholine challenge test. **d** The number of inflammatory cells in BALF of mice with different treatment. **e** The expression of IL-4, IL-5, and IL-13 in BALF of mice with different treatments as determined by ELISA. **f** The expression of α-SMA determined to measure the area of BSMCs in the lung tissues as examined by immunohistochemistry. *p < 0.05 vs. sh-NC. #*p* < 0.05 vs. sham-operated mice. These data were measurement data, expressed as mean ± standard deviation. Data among multiple groups were compared using one-way ANOVA, combined with Tukey’s post-hoc tests. Repeated measures ANOVA was performed for comparison on data at different time points among each group, combined with Bonferroni *post-hoc* tests. n = 6

In this part of the study, we constructed a mouse model of asthma induced by OVA and injected sh-HDAC4 and CXCL12 lentiviruses into the model mice via a tail vein. The OVA-induced mice were injected with sh-NC, sh-HDAC4, sh-HDAC4 + vector, or sh-HDAC4 + CXCL12. RT-qPCR showed that, relative to sham-operated mice, the expression of HDAC4, KLF5, Slug, and CXCL12 in OVA was increased, but that this increase was neutralized by sh-HDAC4. Compared with sh-HDAC4 and vector treatment, sh-HDAC4 and oe-CXCL12 treatment did not change the expression of HDAC4, KLF5, and Slug in mouse lung tissues, but the expression of CXCL12 was increased (Fig. 6a). Western blot assay showed that the expression of HDAC4, Slug, and CXCL12 was upregulated by OVA treatment, accompanied by downregulated KLF5 and Ac-KLF5. sh-HDAC4 brought about significant decreases in the expression of HDAC4, Slug, and CXCL12, but increases in KLF5 and Ac-KLF5 expression. Relative to sh-HDAC4 and vector treatment, sh-HDAC4 and oe-CXCL12 treatment resulted in no changes in the expression of HDAC4, KLF5, Ac-KLF5, and Slug, while increasing CXCL12 expression (Fig. 6b).

The methacholine challenge test displayed that, compared with sham-operated mice, the OVA-induced asthmatic mice had increased airway hyperresponsiveness, indicating successful of modeling. sh-HDAC4 inhibited the AHR in mice caused by OVA, but this inhibition was reversed upon sh-HDAC4 and oe-CXCL12 treatment (Fig. 6c). Moreover, the number of inflammatory cells in BALF of OVA-induced asthmatic mice was higher than that in BALF of sham-operated mice. The increased number of inflammatory cells in mouse BALF caused by OVA induction was reduced by sh-HDAC4. Relative to sh-HDAC4 and vector treatments, sh-HDAC4 and oe-CXCL12 treatment increased the number of inflammatory cells in mouse BALF (Fig. 6d). Based on the results from ELISA, the expression of IL-4, IL-5, and IL-13 in mouse BALF was increased after OVA induction, but was decreased after treatment with sh-HDAC4. In comparison to sh-HDAC4 and vector treatment, the expression of IL-4, IL-5, and IL-13 in mouse BALF showed increases in the presence of sh-HDAC4 and oe-CXCL12 treatment (Fig. 6e).

The results from immunohistochemistry showed that, in comparison to sham-operated mice, OVA-induced asthmatic mice displayed increased expression of α-SMA, which was diminished upon the further treatment of sh-HDAC4 (Fig. 6f). Taken together, HDAC4 promoted airway inflammation and remodeling in asthmatic mice by regulating KLF5/Slug/CXCL12 axis.

## Discussion

Asthma is a highly prevalent chronic lower respiratory disorder presenting with a rising incidence [23]. The treatment of asthma is still clinically challenging due to poorly controlled symptoms or aggravation [24]. In the current study, we explored the contributory role of HDAC4-mediated KLF5/Slug/CXCL12 axis in the development of asthma.

Initially, we identified an upregulated HDAC4 expression in lung tissues of OVA-induced asthmatic mice, and showed that its inhibition alleviated airway inflammation and remodeling. In line with these findings, downregulated HDAC4 by hsa-miR-20a-5p has previously shown to aid in reducing allergic inflammation in HMC-1 cells [9]. Increased HDAC4 expression could accelerate ASMC proliferation by elevating cyclin D1 protein expression [8]. Moreover, the suppression of HDAC4 could participate in a miR-220-mediated self-defense mechanism against abnormal epithelial responses in asthma [25]. Similarly, upregulated HDAC4 expression has been found in the main respiratory muscle of patients with chronic obstructive pulmonary disease [26].

In addition, we found that HDAC4 deacetylated KLF5 to promote its transcription in asthma. In line with our finding, previous work shows that HDAC can deacetylate KLF5 through proteasomal degradation [10]. In addition, HDAC2 could cause Klf5 deacetylation in vascular smooth muscle cells [27]. Thus, as previously reported, KLF5 serves as an inducer of respiratory diseases. For instance, KLF5 was found to accelerate the inflammation of human bronchial epithelial cells BEAS-2B and HBE135-E6E7 as well as to facilitate the proliferation and migration of BSMCs, thereby promoting the progression of asthma [13]. KLF5 displayed upregulation in chronic obstructive pulmonary disease tissues, and downregulated KLF5 expression by miR-145-5p could exert protection against cigarette smoke extract-induced airway epithelial cell apoptosis as well as inflammation [28]. Additionally, KLF5 was revealed to be not only upregulated in small airways and pulmonary vessels of chronic obstructive pulmonary disease patients, but was also implicated in the remodeling of chronic obstructive pulmonary disease tissues [29]. We thus conclude that the interaction between HDAC4 and KLF5 through deacetylation plays an important role in asthma development.

Furthermore, we demonstrated that KLF5 bound to the promoter of Slug to increase its expression in asthma, thereby reducing airway inflammation and remodeling. Previous studies have unveiled the regulation of KLF5 on Slug. For instance, KLF5 was revealed to promote the transcription of Slug in a direct manner, which promoted mammary stemness [14]. Moreover, silencing of KLF5 could downregulate the protein expression of Slug in Hep-2 cells [15]. Intriguingly, increased Slug expression by DP2 in part triggered epithelial-mesenchymal transition involving airway remodeling [30]. Downregulation of Slug by resveratrol treatment attenuated airway inflammation and structural changes in OVA-induced asthmatic mice, in part by reducing airway hyperresponsiveness, inflammation, and α-SMA expression [16]. In the present study, we further extended the mechanistic exploration, and found that Slug could upregulate CXCL12 to promote the inflammation in bronchial epithelial cells and thus enhance the proliferation and migration of BSMCs. In previous work, the neutraligand of CXCL12 was reported to reduce dendritic cell infiltration into the airways and to diminish airway eosinophilic inflammation in OVA-challenged asthmatic mice [31]. Furthermore, CXCR4 was reported to decrease airway responsiveness and inflammation by reducing the levels of IL-4, IL-5, and IL-13 in the BALF in OVA-induced asthma [32]. Notably, decreased CXCL12 expression was observed in S100β-positive dendritic (?) cells after silencing of Slug [33]. Slug was also found to enhance migration and invasion of prostate cancer cells by activating the CXCR4/CXCL12 axis [34]. In addition, knockdown of the receptor of CXCL12, namely, CXCR4, downregulated mesenchymal markers including Slug in intrahepatic cholangiocarcinoma [35]. Taken together, we demonstrated in this study that the KLF5/Slug/CXCL12 axis facilitates the proliferation and migration of BSMCs and the airway inflammation and remodeling in asthmatic mice. Meanwhile, other important mediators and signaling pathways have been noted in the literature, including the crosstalk between HMGB1, Hsp72 and RAGE/ERK1/2 signaling, which is a metabolic pathway common to obesity and bronchial asthma [36].

## Conclusion

**Fig. 7:**
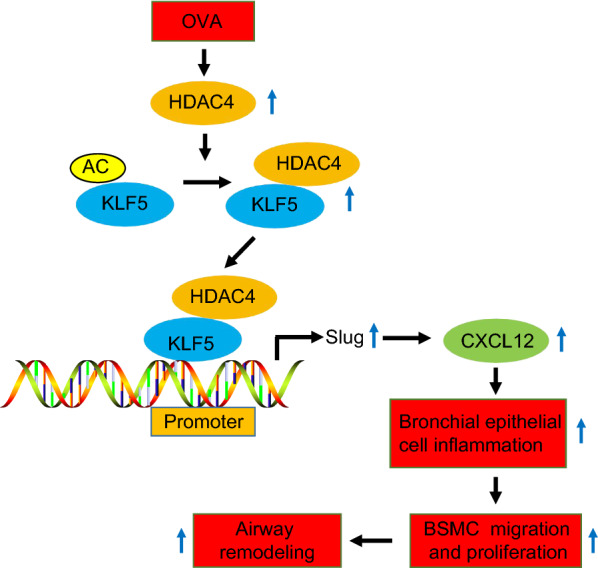
Molecular mechanism regarding HDAC4-mediated KLF5/Slug/CXCL12 axis in asthma. HDAC4 deacetylates KLF5 and increases its transcriptional activity. The deacetylated KLF5 promotes the expression of Slug in the promoter region of Slug, and then promotes the expression of CXCL12. This causes inflammation in bronchial epithelial cells and then induces the proliferation and migration of BSMCs, which leads to airway remodeling and thus aggravates the progression of asthma in mice

In summary, HDAC4 deacetylates KLF5 and increases its transcriptional activity to promote the expression of Slug and further upregulate CXCL12 expression, which causes the inflammation of bronchial epithelial cells and then induced the proliferation and migration of BSMCs, leading to airway remodeling, and thus facilitating the progression of asthma (Fig. 7). This finding unfolded a new mechanism involving HDAC4 in the occurrence of asthma, which requires further verification.

## Supplementary Information

**Additional file 1: Table S1. **Primer sequences for RT-qPCR.

## Data Availability

The datasets generated/analyzed during the current study are available.

## Abbreviations

AHR: Hyperresponsiveness
BSMCs: Bronchial smooth muscle cells
HDAC4: Histone deacetylase 4
KLF5: Kruppel-like factor 5
CXCL12: CXC chemokine ligand-12
PBS: Phosphate buffer saline
BALF: Bronchoalveolar lavage fluid
ELISA: Enzyme-linked immunosorbent assay
OD: Optical density
HE: Hematoxylin–eosin
IP: Immunoprecipitation
CCK-8: Cell-counting kit-8
TGF-β: Transforming growth factor-β
ASMCs: Airway smooth muscle cells
α-SMA: Alpha smooth muscle Actin
IL-4: Interleukin 4
