# Supplementary material for: HDAC4 induces the development of asthma by increasing Slug-upregulated CXCL12 expression through KLF5 deacetylation
Source: J Transl Med. 2021 Jun 12;19:258. doi: 10.1186/s12967-021-02812-7 (PMC8199843; doi:10.1186/s12967-021-02812-7)
Supplement: Supplementary file 1 — Additional file 1: Table S1. Primer sequences for RT-qPCR. [file 12967_2021_2812_MOESM1_ESM.docx]

**Table S1** Primer sequences for RT-qPCR

| Gene | Primer sequences |
| --- | --- |
| hsa-HDAC4 | F: 5'-TCAGATCGCCAACACATTCG-3' |
|  | R: 5'-ACGGGAGCGGTTCTGTTAGA-3' |
| hsa-KLF5 | F: 5'-ACACCAGACCGCAGCTCCA-3' |
|  | R: 5'-TCCATTGCTGCTGTCTGATTTGTAG-3' |
| hsa-Slug | F: 5'-ATGCCGCGCTCCTTCCT-3' |
|  | R: 5'-TGTGTCCAGTTCGCT-3' |
| hsa-CXCL12 | F: 5'-TGCCAGAGCCAACGTCAAG-3' |
|  | R: 5'-CAGCCGGGCTACAATCTGAA-3' |
| hsa-GAPDH | F: 5'-TGCACCACCAACTGCTTAGC-3' |
|  | R: 5'-GGCATGGACTGTGGTCATGAG-3' |
| mmu-HDAC4 | F: 5'-TGGTGTTGGGGTGGATAGCG-3' |
|  | R: 5'-TGGGGTCATTGTAGAAGGCC-3' |
| mmu-KLF5 | F: 5'-CCATTTTCAGCCACCAGAGCGAGT-3' |
|  | R: 5'-ACGGGGGAAAATAAGTGGCCTGCT-3' |
| mmu-Slug | F: 5'-GCTCCTTCCTGGTCAAGAAACAT-3' |
|  | R: 5'-CCGAGGTGAGGATCTCTGGTT-3' |
| mmu-CXCL12 | F: 5'-GAGCCAACGTCAAGCATCTG-3' |
|  | R: 5'-CGGGTCAATGCACACTTGTC-3' |
| mmu-GAPDH | F: 5'-GGCAAAGTGGAGATTGTTGC-3' |
|  | R: 5'-AGCTCTGGGATGACCTTGC-3' |

Note: GAPDH, glyceraldehyde-3-phosphate dehydrogenase; HDAC4, histone deacetylase 4; KLF5, Kruppel-like factor 5; CXCL12, CXC chemokine ligand-12; RT-qPCR, reverse transcription quantitative polymerase chain reaction.
